# Exploring the Relationship Between Ethical Leadership and Nurses' Moral Courage in China: The Mediating Effect of Psychological Empowerment

**DOI:** 10.1155/2024/6664191

**Published:** 2024-10-10

**Authors:** Xuan Wang, Lijuan Zhou, Xianxiu Wen, Li Gou

**Affiliations:** ^1^Department of Nursing, Sichuan Provincial People's Hospital, University of Electronic Science and Technology of China, Chengdu, China; ^2^Department of Nursing Research Centre, Sichuan Provincial People's Hospital, University of Electronic Science and Technology of China, Chengdu, China

**Keywords:** ethical leadership, moral courage, nurses, psychological empowerment

## Abstract

**Aim:** To empirically investigate the impact of ethical leadership on nurses' moral courage in China and examine the mediating role of psychological empowerment in this relationship.

**Background:** Moral courage is essential for alleviating nurses' moral distress, safeguarding patients' safety and rights, and providing high-quality care. Previous studies have emphasized the strong relationship between ethical leadership and moral courage; however, little is known about the actual impact of ethical leadership on nurses' moral courage. This study introduces psychological empowerment, sets out to test empirically its role in the relationship between ethical leadership and moral courage, and provides countermeasures and a theoretical basis for cultivating nurses' moral courage.

**Methods:** Between February and May 2023, a questionnaire survey was conducted among 837 nurses from five hospitals in Sichuan Province, southwest China. The three validated self-report scales, the ethical leadership scale, psychological empowerment scale, and nurses' moral courage scale, were used to collect data. We used IBM SPSS 27.0 for descriptive statistics, univariate analyses, Cronbach's *α*, and correlations of each variable, modeling a hypothesized model of ethical leadership on moral courage in nurses and testing the internal mechanisms by AMOS 26.0.

**Results:** Ethical leadership significantly positively correlated with psychological empowerment (*r* = 0.374, *p* <  0.01), as well as moral courage (*r* = 0.341, *p* <  0.01). In addition, psychological empowerment significantly positively correlated with moral courage (*r* = 0.518, *p* <  0.01). The structural equation modeling (SEM) shows a satisfactory model fit: *χ*^2^ = 2156.36, *df* = 849, *χ*^2^/*df* = 2.540, root mean square of approximation = 0.043, standard root mean square residual = 0.047, normed fit index = 0.911, Tucker–Lewis index = 0.941, and comparative fit index = 0.944. Ethical leadership directly influenced moral courage (*β* = 0.135, *p*=0.006) and indirectly affected moral courage via psychological empowerment (*β* = 0.247, *p*=0.001).

**Conclusion:** Promoting ethical leadership and psychological empowerment is essential for nurses to promote moral courage. The results of this study illustrate the pivotal role of psychological empowerment in establishing the impact of ethical leadership on nurses' moral courage and the partially mediating part of psychological empowerment in this relationship.

**Implications for Nursing Management:** Nursing managers should understand the importance of moral courage in protecting patients' rights and interests, as well as in maintaining a stable nursing workforce. They should reinforce the positive influence of ethical leadership and embrace an ethical leadership style. In addition, providing relevant training to enhance nurses' psychological empowerment will help cultivate moral courage among them.

## 1. Introduction

As direct providers of healthcare services, nurses play an essential role in maintaining human health and the development of healthcare. In 2023, the National Health Commission of China has clearly emphasized the need to improve nursing services, ensure nursing quality and safety, and safeguard people's health rights and interests [1]. From a humanist perspective, nursing is an ethical practice that requires nurses to have the courage to take a moral stance and remain confident in what they believe is right. However, due to the complexity of the clinical environment and other issues, as with some other developing countries [2, 3], Chinese nurses often encounter ethical dilemmas with hospital organizations, managers, colleagues, and patients, such as limited human resources, staffing insufficient to provide quality care to patients, provide “active care” to patients that have no therapeutic effect or does not remove the patient from life support dependency, some ethical dilemmas of care due to patients' financial constraints, and the malpractice of physician and colleague, which are making them in ethical dilemmas, generating negative emotions and burnout, and even hindering the development of the healthcare system [4]. Previous research has shown that nurses with moral courage are better able to deal with ethical issues and experience fewer ethical dilemmas [5] and have the ability to overcome their weakness of will when faced with situations that conflict with their professional values to take action to face ethical challenges head-on. Accordingly, studying the moral courage of nurses is of great significance in improving the quality of nursing services and safeguarding patients' rights and interests.

## 2. Literature Review

Moral courage is recognized globally as crucial to promoting patient safety and ethical care [6], and the core is safeguarding patients' rights [7]. As a fundamental virtue, moral courage refers to a nurse acting under pressure and following their work situation and professional orientation, taking responsibility for decision-making without fear of danger, and rational acting despite expected adverse consequences [8]. Moral courage plays a pivotal role in improving the psychological resilience of nurses and the patient's quality of life [9]. It inspires nurses to advocate for patients when their rights are threatened [10]. The main personal characteristics of nurses' moral courage include moral integrity, acting according to one's conscience, accountability, and self-actualization [8]. Previous studies on moral courage focus on measuring its levels and comparison across groups such as gender, age, educational status, and education related to medical ethics [9, 11], these do not address the mechanisms that influence moral courage. Further studies found that nurses rated their moral courage as high and considered themselves morally courageous but found it difficult to be truly courageous when faced with ethical dilemmas in practice, and still needed support from their organizations and superiors [9]. Organizational factors have been identified as influences on nurses' moral courage [12]; however, leadership as an essential factor in organizational contexts has rarely been explored.

Brown defines ethical leadership as demonstrating normatively appropriate conduct through personal actions and interpersonal relationships and promoting such behavior to followers through two-way communication, reinforcement, and decision-making [13]. In the field of nursing, nursing managers with ethical leadership support the development of moral competence in nurses as pioneers in the nursing profession who are role models for nurses and their behaviors will influence the ethical conduct of nurses [14]. Ethical leadership attempts to create and maintain a favorable ethical climate in the work environment [15]. It is thought to be an effective leadership style in nursing management. Combing through the relevant literature revealed that studies exploring ethical leadership in nursing have yielded positive results. A study by El-Gazar et al. has shown that psychological safety mediated the link between ethical leadership and nurses' internal whistleblowing intentions, and nurse leaders can foster nurses' intentions to blow the whistle internally by adopting ethical leadership behaviors and enhancing psychological safety among nurses [16]. In addition, nurses with a high level of flourishing and who work with a manager exhibiting ethical leadership behavior are more likely to activate extra-role behavior to go the extra mile in their jobs [17]. However, moral courage, a well-recognized virtue in nursing, has not yet been the subject of a study focusing on the positive effects of ethical leadership on nurses' moral courage in the context of Chinese culture. Therefore, it is necessary to conduct this study to fill the gap in existing research and provide a basis for exploring the theory of intervention strategies for moral courage.

Psychological empowerment is the psychological condition or cognitive sense of being empowered that individuals experience in a specific work situation, which reflects the individual's positive attitude toward their work role [18]. Job meaning, competence, self-efficacy, and impact are the four parts of psychological empowerment that make up an individual's intrinsic motivation to work [19]. Studies have shown that nurses who have higher psychological empowerment can enhance innovative behavior [20], increase job satisfaction [21], and reduce ethical conflicts at work [22]. Moreover, the psychological condition of nurses is also an important factor affecting patient satisfaction [23]. According to previous research reports, Khoshmehr et al. [24] found that the relationship between psychological empowerment and moral courage positively correlates with nurses having a higher level of psychological empowerment and showing more courage in the face of internal fears [25]. In addition, there is a positive relationship between ethical leadership and psychological empowerment [26, 27]. In other words, ethical leadership enables employees to experience meaningful job roles throughout their careers. Although the results of previous studies have confirmed the two-by-two correlation between ethical leadership, psychological empowerment, and moral courage in nurses, no studies have been conducted on the coordinated role of ethical leadership and psychological empowerment together on moral courage in nurses and the mechanism of psychological empowerment in ethical leadership and moral courage in nurses has not been explored.

## 3. Theoretical Framework and Hypotheses

Social learning theory [28] states that personal behavior, especially more complex behavior is learned primarily by observing or imitating the behavior of others. The theory stresses the importance of individual cognition in the learning process and the interconnectedness of cognition and the environment in influencing individual learning to produce or change a behavior. In this study, ethical leadership focuses on ethics, as a leader and role model for nurses, treating nurses with respect and fairness, not only being ethical people themselves but also creating a work environment with an ethical atmosphere for nurses [29], which is an organizational situational factor to which individuals belong; psychological empowerment brings nurses a deeper level of psychological cognition, reflecting the process of personal growth and development, which is the cognitive feeling of individuals in the work environment. Nurses with moral courage dare to speak out for the rights, safety, and justice of patients in the face of conflicts and unfavorable situations that arise in nursing practice [30], which is considered to be an essential characteristic and ethical behavior that nurses should possess.

According to Thomas and Velthouse [31], employee psychological empowerment is shaped and influenced by the work contexts, such as leaders and leaders' behavior. Based on the social learning theory, when nurses feel stimulated by the nurse manager and perceive the role as trustworthy, they are more likely to build up their psychological perceptions and gain psychological empowerment in work by observing and mimicking the attitudes and behaviors of the nurse manager. When nurses have psychological empowerment at work, they are more courageous to take practical actions to face and deal with some ethical issues in their daily work, which positively affects their moral courage. Therefore, based on the social learning theory and other relevant literature reviewed above, this study explores the potential mechanisms among ethical leadership, psychological empowerment, and nurses' moral courage. We propose the four hypotheses as follows:

1. Hypothesis 1: Ethical leadership is positively related to psychological empowerment.
2. Hypothesis 2: Psychological empowerment is positively related to moral courage.
3. Hypothesis 3: Ethical leadership is directly positively related to moral courage.
4. Hypothesis 4: Psychological empowerment mediates the association of ethical leadership with moral courage.

## 4. Methods

### 4.1. Study Design

This cross-sectional study was part of a project aimed at revealing the relationship between ethical leadership, spiritual climate, psychological empowerment, nurse–patient relationship, and nurses' moral courage. A previous article reporting on the relationship between spiritual climate and the psychological empowerment of nurses in China has been published [32]. This article reports findings on the impact of ethical leadership on nurses' moral courage and examines the mediating role of psychological empowerment in this relationship.

### 4.2. Participants and Setting

Sichuan Province is located in the southwestern interior of China and consists of 5 regions: East Sichuan, South Sichuan, West Sichuan, North Sichuan, and Central Sichuan. As reported by Wang et al. [32], a questionnaire survey was conducted from February to May 2023 using the convenience sampling method among registered nurses in five hospitals selected from these five regions, all of which were tertiary care public hospitals with more than 1500 beds. All nurses were from different departments, including internal medicine, surgery, obstetrics and gynecology, pediatrics, emergency, and intensive care unit (ICU). The structural equation model (SEM), with a sample size of at least 200, was recommended [33], or 10 times the number of variables. The number of observed variables for this study was 18; thus, a sample size of 837 nurses aligned with the above rules. The study inclusion criteria for nurses were (1) registered nurses, (2) having at least 1 year of nursing in the current hospital, and (3) voluntary participation in this study. The criteria excluded nurses who were (1) not directly involved in patient care, such as on sick or personal leave and (2) nursing managers at the level of the head nurse and above.

### 4.3. Measurements

#### 4.3.1. Nurse Demographic Characteristics

The research team designed its questionnaire on the demographic characteristics of nurses by reviewing the literature and questioning experts, which included gender, age, work department, years of working, education level, employment type, professional title, training in medical ethics (yes/no), and marital status items.

#### 4.3.2. Ethical Leadership Scale (ELS)

The ELS was created by Brown et al. [13] and designed to measure ethical leadership from nurses' perspectives. Bian [34] translated it into Chinese and validated it. The scale is single-dimensional and consists of 10 items. An example item is “My head nurse sets an example of how to do things correctly regarding ethics.” Each item evaluates from “*strongly disagree* = 1” to “*strongly agree* = 5” with a five-point scale. The total scores on the scale ranged from 10 to 50. High scores indicate high levels of ethical leadership of the head nurses from the nurses' perspective. Cronbach's alpha coefficient for the original scale was 0.95 and 0.925 in this sample.

#### 4.3.3. Psychological Empowerment Scale

The PES, a measure of psychological empowerment, was developed by Spreitzer [18], translated into Chinese, and validated by Sun et al. [35]. The scale contains 12 items with four subscales, including job meaning (3 items), job competence (3 items), self-efficacy (3 items), and job impact (3 items). An example item is “I am confident in my ability to do well in all aspects of my job.” Each item measures “*strongly disagree* = 1” to “*strongly agree* = 5” on a five-point scale. The total scores on the scale ranged from 12 to 60. High scores indicate high perceived psychological empowerment by nurses. Cronbach's alpha coefficient of the four subscales of the original scale was in the range of 0.72–0.86 and 0.830–0.935, respectively, in this sample.

#### 4.3.4. Nurses' Moral Courage Scale (NMCS)

The NMCS measures nurses' moral courage. The original scale of NMCS was developed by Numminen et al. [8]. Wang et al. [36] after obtaining authorization from the authors of the original scale strictly followed Brislin's two-person translation-back-translation model for the Chinese version of the NMCS. Five experts (one professor of nursing, three clinical nursing experts, and one professor of psychology) were hired to culturally adapt the Chinese version of the NMCS to make it suitable for the cultural environment of China and to assess its content validity to form the Chinese version of the NMCS. The Chinese version of the NMCS is consistent with the original scale and includes 21 items of four subscales, including moral integrity (7 items), commitment to good care of patients (5 items), compassion and authentic presence with the patient (5 items), and moral responsibility (4 items). Each item measured from “*does not describe me at all* = 1” to “*describes me very well* = 5” on a five-point scale. The total scores on the scale ranged from 21 to 105. High scores indicate high levels of nurses' moral courage. Cronbach's alpha coefficient of the four subscales of the original scale was in the range of 0.778–0.902 and 0.866–0.907, respectively, in this sample.

### 4.4. Ethical Consideration

The study was approved by the Ethics Committee of the Sichuan Provincial People's Hospital, University of Electronic Science and Technology of China, the first author's institution. Researchers explained the research's aim, significance, and precautions to all participants and distributed the questionnaire only after receiving each participant's informed consent. All data collected were anonymized, and the results of the completed questionnaires were not visible to anyone other than the researcher and the respondents themselves and were used only for the study.

### 4.5. Data Analysis

The descriptive statistics, univariate analyses, Cronbach's *α*, and correlations of each variable used the IBM SPSS 27.0 (IBM, New York, NY, USA) to analyze, using frequency and percentage for count date and mean ± standard deviation (*M* ± SD) for normally distributed measures. Pearson's coefficient was used to examine the correlations among ethical leadership, psychological empowerment, and nurses' moral courage, modeling a hypothesized model of ethical leadership on moral courage in nurses and testing the internal mechanisms by AMOS 26.0 (IBM, New York, NY, USA). The bootstrapping procedure of 5000 resamples tested the significance of the mediating effects model, and the 95% confidence interval without zero considered the mediating effect significant. Differences are two-sided, and a *p* value below 0.05 is considered statistically significant.

## 5. Data Collection

We used paper-based questionnaires for our survey. First of all, we contacted the director of the nursing department of each investigated hospital, explaining in detail the purpose, significance, and content of this study's survey. After obtaining the consent, the nursing department of the investigated hospitals arranged for one person in charge to dock with the researchers. Then, the researchers used a unified instruction to inform the people in a ward of the investigated hospital about this study's purpose, significance, data collection methods, and quality control methods. Finally, the researchers sent the nurses a paper version of the questionnaire to fill in. The questionnaires were filled out during each department's morning shift or group meeting and collected on the spot after completion. Respondents involved in questionnaire completion were (1) informed that participation was voluntary and that they could withdraw at any time without reason; (2) the study was anonymous and the results of questionnaire completion would not be seen by coworkers, leaders, etc.; and (3) the survey information obtained was to be used only for nursing study. In total, 851 nurses completed and returned the questionnaires, of which 14 questionnaires were dropped from the analysis due to incomplete completion, and 837 valid questionnaires were analyzed (response rate: 98.35%).

## 6. Results

### 6.1. Demographic Characteristics of Nurses and the Univariate Analyses of Nurses' Moral Courage

Among 837 nurses, 94.1% were female, 80.9% had a bachelor's degree, 30.7% had a work experience of 6∼10 years, and 69.2% were married. In addition, univariate analyses showed significant differences in gender, age, departments, years of working, professional titles, and training in medical ethics (yes/no) and marital status showed significant differences in nurses' moral courage scores. The details are given in Table 1.

### 6.2. The Score of Nurses' Moral Courage in the Current Study

The total scores (*M* ± SD) of nurses' moral courage in Sichuan Province, southwest China, was 80.68 ± 12.04, which is mainly moderate to high, and the scores of subscales are detailed in Table 2.

### 6.3. Correlations for Variables of the Study

Pearson's correlation analysis showed that ethical leadership and psychological empowerment were positively correlated (*r* = 0.374, *p* <  0.01), as were moral courage (*r* = 0.341, *p* <  0.01). In addition, positive correlations between psychological empowerment and moral courage can be found (*r* = 0.518, *p* <  0.01), and the relationships between each scale subscale are detailed in Table 3.

### 6.4. SEM of Study Variables

**Figure 1:**
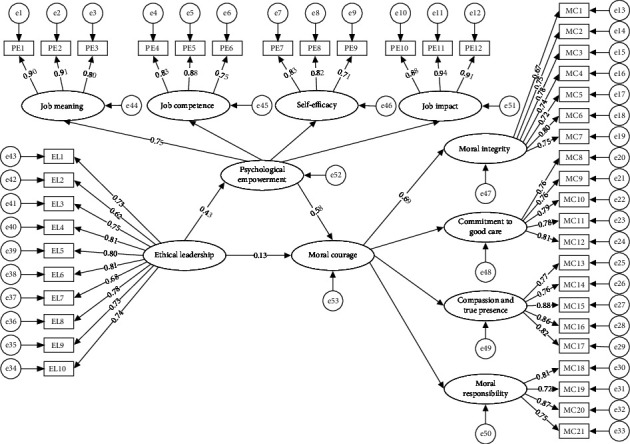
The final model of moral courage among nurses (with standardized regression coefficients). ^∗∗^*p* < 0.01. EL1–EL10: items of ethical leadership; PE1-PE12: items of the four dimensions of psychological empowerment; MC1–MC21: items of the four dimensions of moral courage.

As shown in Table 4, the model offers a satisfactory model fit: *χ*^2^ = 2156.36, *df* = 849, *χ*^2^*/df = *2.540, RMSEA = 0.043, SRMR = 0.047, NFI = 0.911, TLI = 0.941, and CFI = 0.944, and all indicators met the requirements. As shown in Figure 1 and Table 5, the mediating effect value of psychological empowerment is 0.247, which accounts for 64.8% of the total effect value of 0.381. Psychological empowerment partially mediates the relationship between ethical leadership and nurses' moral courage.

## 7. Discussion

This study explored the relationship among ethical leadership, psychological empowerment, and moral courage from nurses' perspectives, established the impact of ethical leadership on nurses' moral courage, and examined the partially mediating role of psychological empowerment in this relationship. To the best of our knowledge, this study is the first to investigate these associations and explore the potential mechanisms.

The findings of this study showed that the total scores of moral courage in nurses are moderate to high, which is analogous to other studies [37, 38]. It indicates that despite the complex and challenging healthcare environment, nurses are willing to provide good nursing care to patients and advocate for protecting their rights and interests. In this study, the highest score (3.99 ± 0.72) for moral responsibility indicated that nurses possess essential ethical qualities and can deal with ethical challenges at work with practical actions. The sample of nurses in this study were all from public tertiary-level hospitals in Sichuan Province, which, along with the high-quality development of public hospitals, requires nurses to possess professional knowledge and the ability to provide optimal solutions for patients when they face compromised rights and interests. The lowest commitment to good care of patients score (3.73 ± 0.78) indicates that in practice, nurses may compromise on protecting the rights of patients and remain silent on ethical issues that arise in the care process, and the reasons for this may be analyzed to be related to insufficient training in this area, lack of supportive managers, fear of adverse consequences due to the fear of risk-taking and bullying, and loss of job [39]. Moral courage as a moral virtue enables nurses to make the right decisions and behaviors when faced with ethical distress and helps nurses deal with ethical challenges with aplomb [40]. It is recommended that hospitals and administrators create a work environment that promotes teamwork and respectful communication. Clinical nurses should practice a “patient-centered” orientation, have solid nursing expertise and skills, understand their realities, and provide nursing care and services guided by evidence-based practice theories and nursing quality standards. Only then is it possible to gain the respect and recognition of colleagues, patients, and families when faced with difficult clinical decisions.

The results of the univariate analysis revealed that nurses' moral courage scores varied by gender, age, work department, years of working, professional title, whether or not had received medical ethics training, and marital status. In this study, the moral courage of male nurses was lower than that of female nurses, and one possible explanation is that under the influence of traditional concepts in Chinese society, male nurses not only suffered from prejudice and discrimination from society, patients, and family members but also faced hostility from within the healthcare workforce [41], which greatly affected their enthusiasm for nursing, and thus male nurses mostly adopted the negative strategy of avoidance in the face of the reality of ethical dilemmas. Older nurses, who have more years of working, and have higher professional titles, show more morally courageous behaviors, which is largely similar to the findings of existing studies [12, 38], and this may be because younger nurses usually undertake routine and general patient care, lack experience of moral courageous actions, and become overwhelmed when encountering situations triggering ethical distress; similarly, nurses with higher job titles tend to undertake more specialized and skilled care of acute and critical patients, and as their work experience grows, individuals become more aware of special situations and able to recognize behaviors and are more comfortable in dealing with ethical dilemmas or conflicts. ICU is the setting where moral dilemmas and challenges most often arise, and previous studies have shown that ICU nurses have higher levels of moral courage [42, 43]. However, in this study, nurses working in ICUs showed lower levels of moral courage, the reason for this may be analyzed to be related to the different time points of data collection, as the survey sample of this study was collected after the opening of the new coronary pneumonia epidemic, whereas the samples of the other studies originated from the period of the new coronary pneumonia pandemic, during which time, admissions to the ICUs in all hospitals increased dramatically, and the nurses would have been faced with ethical dilemma situations more frequently. This is consistent with Numminen's [44] conceptual analyses of moral courage which suggest that “experience” is a prerequisite for the development of moral courage. In addition, the moral courage of nurses who had received medical ethics training was higher than those who had not, which is consistent with a study by DeSimone [45] regarding the positive effect of ethical knowledge base, ethics training, and education on the level of moral courage. Finally, the results of this study also showed that marital status may also be a factor influencing moral courage among nurses, and unmarried nurses had lower moral courage scores than married, the reason for this may be that unmarried nurses have a lower level of social support, receive less emotional support, and are more isolated, which may affect their courage to speak up and act courageously in situations filled with challenging ethical dilemmas. These indicate that managers should pay more attention to the work status of junior nurses, nurses working in ICUs, and single nurses; support them to be able to bravely express their views when they encounter ethical dilemmas at work; and respect their ethical decision-making while ensuring that patients' rights and interests are not compromised. As a component of nurses' moral competence, moral courage is a personal characteristic that can be learned and developed, and hospital administrators should include training courses on moral courage in their daily training to encourage nurses to play the role of moral courage in practice and bravely carry out their obligations to protect patients. Some countries have included moral courage training courses in their teaching curricula with more positive results, and their training methods are worth exploring and practicing.

Our study shows that ethical leadership has a positive impact on nurses' moral courage, consistent with several existing studies suggesting a significant positive relationship between ethical leadership and moral courage [46, 47], nurses perceive a higher level of ethical leadership from nurse managers, and more moral courage they are inspired to have Traditional Chinese culture often emphasizes the differences between the upper and lower levels and the order of respect and inferiority, with leaders often occupying a more crucial dominant position in an organization and having a more prominent influence on their subordinates. When nurse leaders demonstrate ethical leadership behaviors that are more focused on the work process, the interests of nurses, and doing the right thing, they are more effective in inspiring the formation of moral courage in nurses. In addition, the present result supports the previous finding of a positive correlation between ethical leadership and psychological empowerment [27]. Psychological empowerment is a motivational state that is achieved through a combination of job meaning, autonomy, self-efficacy, and perceived impact of work. It has a positive orientation towards employees' work so that they are interested and feel empowered to shape their work environment. Ethical leadership often takes into account the growth and developmental needs of each nurse, motivates nurses to perform core job duties, and puts them in a position where they can experience appropriate and meaningful work roles in their careers, thereby influencing their psychological empowerment and motivating them to be proactive rather than passive in their work. Finally, this study showed a positive relationship between psychological empowerment and moral courage. When nurses have high levels of psychological empowerment, they will be more likely to demonstrate higher levels of morally courageous behavior. Malak et al. found that nurses with high levels of psychological empowerment believe in their ability to do all things well at work, are less psychologically stressed in the work environment, and are more motivated to serve their patients [48]. Thus, it can be seen that having a high level of psychological empowerment will motivate nurses to comply with the code of ethics and still choose to protect patient's rights and interests when their legitimate rights and interests are compromised, without fear of being threatened, fearful, or even losing their jobs.

The results of SEM showed that ethical leadership had a direct effect on nurses “moral courage (95% CI excluding 0, *p*  <  0.05), and ethical leadership could also have an indirect effect on nurses' moral courage through psychological empowerment (95% CI excluding 0, *p*  <  0.05), and that psychological empowerment partially mediated the relationship between ethical leadership and nurses' moral courage, which support Hypothesis 4 of the study. The results of this study show that ethical leadership directly and positively influences nurses' moral courage, with the direct effect accounting for 35.2% of the total effect. According to social learning theory, personal behaviors, especially more complex behaviors, can be acquired by individuals by observing and learning to imitate the behaviors of their role models; however, the type of behaviors accepted and the effectiveness of their implementation depend on the role of the role model. Previous research has shown that leader style is the most important situational factor influencing employees' moral courage [49]. As leaders and role models for nurses, ethical leaders respect nurses, handle all matters at work with fairness and integrity, and encourage nurses to speak up for patient rights when faced with situations that undermine patient rights and interests in nursing practice, which in turn leads to the conscious occurrence of moral courage among nurses. Therefore, nursing managers should strengthen their ethical leadership construction, set an example in their work, and play an exemplary role; for hospital organizations, they should establish a correct orientation for selecting and employing people, and pay attention to selecting and employing managers with moral leadership qualities.

Our study found that the influence of ethical leadership on nurses' moral courage can also be indirectly influenced by psychological empowerment, i.e., ethical leadership can promote nurses' psychological empowerment and then stimulate nurses' moral courage, and the size of the mediating effect of psychological empowerment between the two is 0.247, and the indirect effect accounts for 64.8% of the total effect, suggesting that psychological empowerment plays an important role between ethical leadership and nurses' moral courage. This suggests that moral leadership plays an important role in the relationship between ethical leadership and the moral courage of nurses. Ethical leadership makes nurses feel stronger psychological empowerment in their work so that they dare to do the right thing in favor of patients' rights and interests when they encounter situations that are contrary to patients' rights and interests in their practice. Therefore, to improve the moral courage of nurses, it is important to improve the level of ethical leadership of managers and the nurses' psychological empowerment. The results of this study support the relationships and mechanisms proposed in the social learning theory model, whereby a combination of personal cognition and the environment influences the behavior of the learning individual. Ali Awad and Al-Anwer Ashour [50] also reached a similar conclusion, in whose research, ethical leadership affected moral courage through ethical climate as a mediating variable. Based on the above discussion, intervening in nurses' moral courage from the perspective of psychological empowerment may yield better results. It suggests that nursing managers should integrate ethical awareness and ethical behaviors into the daily work of clinical nurses to enhance the intrinsic motivation and sense of the meaning of nurses' work, which will, in turn, promote the formation of morally courageous behaviors.

## 8. Limitations

This study has some limitations to take into account. First, the study was only conducted in Sichuan Province, southwest China, with limited geographic areas and hospitals involved, therefore, the results obtained need to be validated more extensively in other regions or countries. Second, it may have resulted in reporting potential bias since we relied on self-report questionnaires, there may be a self-glorification of the investigator. Furthermore, the study used convenience sampling, and despite an adequate sample size, the number of male nurses in our study was too small, which may have contributed to selection bias. Finally, the cross-sectional design demonstrates that a causal relationship is not possible, and future research could try to conduct qualitative and longitudinal studies.

## 9. Conclusions

This study explored the correlation between nurses' perceived ethical leadership, psychological empowerment, and moral courage. As expected, ethical leadership and psychological empowerment influenced moral courage among nurses, and psychological empowerment partially mediated their relationship. Nursing managers must adequately recognize the link between these variables and adopt an ethical leadership approach to increase nurses' psychological empowerment and improve their moral courage.

## 10. Implications for Nursing Management

The results of this study show that the moral courage of nurses is moderately high, and ethical leadership and psychological empowerment positively influence it. We suggest hospital administrators pay attention to developing moral courage in nurses by emphasizing that ethics is integral to nursing practice. For example, training on moral courage as part of the nursing ethics curriculum and providing nurses with knowledge of moral courage. Similarly, nursing management should make a prerequisite to understand the meaning of ethical leadership and to identify with the sense of ethical leadership, take the initiative to learn the relevant theoretical knowledge of leadership and ethics, enhance the knowledge and awareness of the ethical leadership style, and create a good working environment and ethical atmosphere so that nurses can obtain psychological empowerment, thus stimulating the potential for the formation of moral courage in nurses.

## Figures and Tables

**Table 1:** Nurse's demographic characteristics and differences in moral courage.

**Variables**	***n* (%)**	**Nurses' moral courage scale**	
		**Mean (SD)**	***t/F***
Gender			−2.063^∗^
Male	49 (5.9)	3.68 ± 0.49	
Female	788 (94.1)	3.85 ± 0.58	
Age			3.937^∗^
18∼25	105 (12.5)	3.81 ± 0.58	
26∼35	497 (59.4)	3.80 ± 0.59	
36∼45	174 (20.8)	3.89 ± 0.53	
> 45	61 (7.3)	4.05 ± 0.44	
Work department			4.429^∗^
Internal medicine	265 (31.7)	3.86 ± 0.60	
Surgery	183 (21.9)	3.90 ± 0.55	
Obstetrics and gynecology	31 (3.7)	3.79 ± 0.61	
Pediatrics	78 (9.3)	3.76 ± 0.56	
Emergency	60 (7.2)	3.79 ± 0.49	
ICU	87 (10.4)	2.60 ± 0.57	
Others	133 (15.8)	3.84 ± 0.57	
Years of working			5.700^∗^
1∼5	205 (24.5)	3.77 ± 0.57	
6∼10	257 (30.7)	3.84 ± 0.60	
11∼15	213 (25.4)	3.79 ± 0.58	
> 15	162 (19.4)	4.00 ± 0.50	
Education level			1.035
Junior college	138 (16.5)	3.87 ± 0.56	
Bachelor's degree	677 (80.9)	3.84 ± 0.57	
Master's degree or above	22 (2.60)	3.68 ± 0.65	
Employment type			−1.568
Formal	148 (17.7)	3.91 ± 0.53	
Contracted	689 (82.3)	3.83 ± 0.59	
Professional title			8.753^∗^
Junior	459 (54.8)	3.81 ± 0.61	
Middle	319 (38.1)	3.83 ± 0.53	
Senior	59 (7.1)	4.14 ± 0.43	
Training in medical ethics			−4.701^∗^
Yes	596 (71.2)	3.90 ± 0.56	
No	241 (28.8)	3.70 ± 0.57	
Marital status			3.534^∗^
Single	226 (27.0)	3.78 ± 0.57	
Married	579 (69.2)	3.86 ± 0.57	
Widowed or separated	32 (3.8)	4.04 ± 0.56	

^∗^ *p* < 0.05.

**Table 2:** The scores of nurses' moral courage in the current study (*n* = 837).

**Variables**	**Items**	**Scores (*M* ± SD)**	**Means (*M* ± SD)**
Moral courage scale	21	80.68 ± 12.04	3.84 ± 0.57
Moral integrity	7	26.53 ± 5.06	3.79 ± 0.72
Commitment to good care of patients	5	18.64 ± 3.89	3.73 ± 0.78
Compassion and true presence with patient	5	19.56 ± 3.55	3.91 ± 0.71
Moral responsibility	4	15.94 ± 2.87	3.99 ± 0.72

**Table 3:** Correlation analysis of ethical leadership, psychological empowerment, and moral courage (*n* = 837).

**Variables**	**1**	**2**	**3**	**4**	**5**	**6**	**7**	**8**	**9**	**10**	**11**
(1) Ethical leadership scale	1										
(2) Psychological empowerment scale	0.374^∗∗^	1									
(3) Job meaning	0.354^∗∗^	0.727^∗∗^	1								
(4) Job competence	0.274^∗∗^	0.746^∗∗^	0.601^∗∗^	1							
(5) Self-efficacy	0.286^∗∗^	0.778^∗∗^	0.449^∗∗^	0.593^∗∗^	1						
(6) Job impact	0.225^∗∗^	0.732^∗∗^	0.265^∗∗^	0.253^∗∗^	0.381^∗∗^	1					
(7) Moral courage scale	0.341^∗∗^	0.518^∗∗^	0.426^∗∗^	0.431^∗∗^	0.387^∗∗^	0.333^∗∗^	1				
(8) Moral integrity	0.302^∗∗^	0.421^∗∗^	0.331^∗∗^	0.328^∗∗^	0.331^∗∗^	0.283^∗∗^	0.821^∗∗^	1			
(9) Commitment to good care	0.195^∗∗^	0.310^∗∗^	0.287^∗∗^	0.293^∗∗^	0.198^∗∗^	0.181^∗∗^	0.743^∗∗^	0.437^∗∗^	1		
(10) Compassion and true presence	0.279^∗∗^	0.483^∗∗^	0.382^∗∗^	0.412^∗∗^	0.365^∗∗^	0.313^∗∗^	0.818^∗∗^	0.517^∗∗^	0.498^∗∗^	1	
(11) Moral responsibility	0.290^∗∗^	0.413^∗∗^	0.343^∗∗^	0.324^∗∗^	0.322^∗∗^	0.265^∗∗^	0.729^∗∗^	0.453^∗∗^	0.376^∗∗^	0.608^∗∗^	1

^∗∗^ *p* < 0.01.

**Table 4:** Model fitting index.

**Statistical test**	**χ** ^2^	***df***	**χ** ^2^ **/*df***	**RMSEA**	**SRMR**	**NFI**	**TLI**	**CFI**
Acceptable threshold	—	—	< 3.00	< 0.08	< 0.05	> 0.90	> 0.90	> 0.90
Hypothesized model	2156.36	849	2.540	0.043	0.047	0.911	0.941	0.944

Abbreviations: *χ*^2^/df = the chi-square degrees of freedom; CFI = comparative fit index; df = degrees of freedom; NFI = normed fit index; RMSEA = root mean square of approximation; SRMR = standard root mean square residual; TLI = Tucker–Lewis index.

**Table 5:** Results of mediation analyses.

**Model path**	**Estimate**	**Se**	***Z***	**95% CI**		**p** ** value**	**Effect ratio (%)**
				**LLCI**	**ULCI**		
*Direct effect*							
EL ⟶ MC	0.134	0.046	2.913	0.040	0.222	0.006	35.2
EL ⟶ PE	0.425	0.041	10.366	0.347	0.510	0.001	
PE ⟶ MC	0.580	0.059	9.831	0.457	0.688	0.001	

*Indirect effect*							
EL ⟶ PE ⟶ MC	0.247	0.036	6.861	0.185	0.326	0.001	64.8
Total effect	0.381	0.040	9.525	0.300	0.459	0.001	

Abbreviations: EL = ethical leadership; MC = moral courage; PE = psychological empowerment.

## Data Availability

The datasets used and analyzed during the current study are available from the corresponding author upon reasonable request.
